# In the Eye of the Beholder? Parent-Observer Discrepancies in Parenting and Child Disruptive Behavior Assessments

**DOI:** 10.1007/s10802-017-0381-7

**Published:** 2018-01-02

**Authors:** Martine A. Moens, Joyce Weeland, Danielle Van der Giessen, Rabia R. Chhangur, Geertjan Overbeek

**Affiliations:** Research Institute of Child Development and Education, University of Amsterdam, Nieuwe Achtergracht 127, 1018 WS Amsterdam, The Netherlands

**Keywords:** Childhood, Externalizing problems, Multi-informant discrepancies, Parenting

## Abstract

This study examined parent-observer discrepancies in assessments of negative child behavior and negative parenting behavior to shed more light on correlates with these discrepancies. Specifically, we hypothesized that informant discrepancy between observers and parents on child behavior would be larger when parents reported high levels of negative parenting (and vice versa) because high levels of these behaviors might be indicators of negative perceiver bias or patterns of family dysfunctioning. Using restricted correlated trait–models, we analyzed cross-sectional observation (coded with the Dyadic Parent-Child Interaction Coding System) and survey data (Eyberg Child Behavior Inventory and Parenting Practices Interview) of 386 Dutch parent-child dyads with children aged 4–8 years (*M*_*age*_ *=* 6.21, *SD* = 1.33; 55.30% boys). Small associations between parent-reported and observed child and parenting behavior were found, indicating high discrepancy. In line with our hypothesis, this discrepancy was higher when parents self-reported more negative parenting or more negative child behavior. Parent-observer discrepancy on negative child behavior was also predicted by child gender. For boys parents reported higher levels of negative child behavior than were observed, but for girls parents reported lower levels of negative child behavior than were observed. These findings suggest that informant discrepancies between observers and parents might provide important information on underlying, problematic family functioning and may help to identify those families most in need of help.

## ᅟ

In assessments of child psychopathology we need multiple informants and methods, especially given the fact that there is no consensus on a ‘gold standard’; a single measure or method that most precisely operationalizes child behavior or functioning (Hunsley and Mash 2007). Because each informant provides unique information, the use of multiple informants has been strongly advocated in the assessment of externalizing or negative child behavior (Dirks et al. 2012; Van der Ende et al. 2012), and negative parenting behavior (Tein et al. 1994). However, a large body of the literature shows that on average there is high discrepancy between informants (e.g., Dirks et al. 2012). The main focus of this discrepancy literature has been on informant discrepancies on negative child behavior between different reporters using questionnaires (i.e., between parent-child, father-mother or parent-teacher), rather than on reporter-observer discrepancies. Moreover, these discrepancies are one of the most poorly understood phenomena in mental health research, making it difficult to decide which informant is of most use when drawing conclusions about child functioning and intervention strategies (Achenbach 2011). Furthermore, by treating informant discrepancy as measurement error we might overlook meaningful information about child and family functioning. This study therefore assessed parent-observer discrepancies on negative child and negative parenting behavior and also endeavored to shed more light on factors associated with these discrepancies.

According to meta-analyses on informant (dis)agreement in the assessment of *child behavior*, agreement levels are small to moderate in strength (Achenbach et al. 1987; Achenbach 2006; De Los Reyes et al. 2015; Duhig et al. 2000; Korelitz and Garber 2016). Discrepancies have been found in a wide range of child behavior reports and they tend to differ in size and direction depending on the assessed behavior, instruments used and informant combinations. For example, small positive correlations were found between parent-child reports on social behavior, whereas moderate positive correlations were found in reports on problem, or negative child behaviors between parent-child or parent-teacher (Gresham et al. 2010; Waaktaar et al. 2005). The largest levels of agreement are found in interparental reports of child externalizing behaviors (Achenbach et al. 1987; Duhig et al. 2000).

Despite the growing body of discrepancy research, only few studies specifically examined levels of agreement between informants on *parenting behavior*. Results of a study that compared parent-child reports of parenting behavior revealed low levels of agreement in parent-child reports on acceptance, rejection, control and inconsistent discipline (Tein et al. 1994). Another study on parent-child reports confirms low to moderate agreement between parent and child on parental monitoring (De Los Reyes et al. 2010). Information on informant discrepancy in the assessment of parenting behavior is of crucial importance, because there might be underlying factors that contribute to informant discrepancies that are associated with specific family dynamics, and parent-child interaction dynamics in particular. For example, adolescents who viewed parenting more negatively than their parents did show higher levels of externalizing behaviors (Dimler et al. 2017). The authors argue that the discrepancy between parent and child might be a proxy for the parent-child relationship.

Moreover, the vast majority of the discrepancy literature has examined informant discrepancies by comparing questionnaire-reports of mothers and fathers (i.e., Duhig et al. 2000), children and their parents (e.g., Reynolds et al. 2011), or teachers and parents (e.g., Harvey et al. 2013). A small amount of studies compared observer reports with parent reports (e.g., Gonzales et al. 1996; Sessa et al. 2001; Swenson et al. 2016), leaving parent-observer discrepancies relatively under-studied. However, observations are commonly used to assess negative child behavior and negative parenting behavior. In a recent meta-analysis, Hendriks et al. (2017) found 37 studies that reported observer ratings and parent ratings on parenting behavior, providing us with new information on parent-observer discrepancies. They found a small (*r* = 0.17), but significant, association between parent-reported and observed parenting. The strength of this association depended on the specific questionnaire used, as well as the specific type of parenting behavior assessed. Stronger associations were found between parent reports and observer reports for negative parenting, whereas smaller associations were found for behavioral control (Hendriks et al. 2017). Despite these previous research findings, we still know relatively little specifically about discrepancies between observers and parents. This study will therefore assess informant agreement between parents and observers, both on negative child behavior and negative parenting behavior, using instruments commonly used in research.

Informant discrepancies are mostly addressed as measurement error or statistical ‘noise’ in previous research. However, more recently it has been argued that such discrepancies might actually be a meaningful phenomenon (see De Los Reyes 2011; De Los Reyes et al. 2013; Hendriks et al. 2017). The Attribution Bias Context Model suggests that informant discrepancies in clinical practice are preceded by differences in informant attributions of the cause of child problem behavior, as well as by differences in informant perspectives in interaction with the context and procedure of assessment (De Los Reyes 2013). Different correlates for informant discrepancies have been suggested, such as socioeconomic factors (Stone et al. 2013), contextual influences (i.e., person-environment interactions: Hartley et al. 2011), and informant characteristics (e.g., parental depression; see Berg-Nielsen et al. 2003; Briggs-Gowan et al. 1996; De Los Reyes et al. 2008, 2011; Guion et al. 2009). For example, emotionally disturbed or depressed parents might report their child’s behavior and/or their own parenting behavior more negatively, because of an overall negativity bias (Haaga et al. 1991, but see for a critical review Richters 1992). Such bias might increase discrepancies between parent-reported and observed behavior. Nevertheless, possible correlates and underlying mechanisms of informant discrepancies –specifically parent-observer discrepancies– are currently still under-studied.

The correlates of informant discrepancy might however also be specific to the psychopathology/behavior assessed (see Martel et al. 2017). In the assessment of negative child behavior and parenting behavior, an important explanation of informant discrepancies might lie in underlying problematic patterns of parent-child interactions and family functioning (e.g., Vierhaus et al. 2016; Dimler et al. 2017). For example, in assessments of negative child behavior, parent-child discrepancies have been related to negative parenting practices (i.e., harsh parenting), parenting stress, and conflictual parent-child interactions (De Los Reyes and Kazdin 2005; Ferdinand et al. 2004; Grills and Ollendick 2003; Stokes et al. 2011; Treutler and Epkins 2003). Although not all studies elaborated on which informant scored higher, in general these studies indicate that parents reported higher levels of child problem behavior than other informants. Parents who experience highly conflictual parent-child interactions might show an increased proneness to report more negatively about child behavior (Grills and Ollendick 2003; Stokes et al. 2011) indirectly leading to higher informant discrepancy. In sum, informant discrepancies in the assessment of negative child and parenting behavior might be associated with dysfunctional family patterns such as coercive parent-child interactions or increasing parenting stress. Identifying possible family factors associated with discrepancies can be specifically valuable for diagnostic and treatment processes (Guion et al. 2009).

### The Present Study

Although the informant discrepancy literature is gaining momentum, a few aspects of this discrepancy in the assessment of child psychopathology are under-studied. First, parent-observer discrepancies might be an important source of information, because they might reveal important information on family functioning. In line with this reasoning, the current study aims to examine discrepancies between parent-reported and observed *negative child behavior* as well as *negative parenting behavior*. Based on the recent Hendriks et al. (2017) meta-analysis, we expect low agreement between observer and parent reports. Acknowledging that discrepancies between informants might reflect underlying problematic family interactions (Ferdinand et al. 2004, 2006; Grills and Ollendick 2002; Guion et al. 2009; Stokes et al. 2011), we expect that discrepancy between parent-reported and observed *negative child behavior* will be larger when parents report relatively high levels of negative parenting (i.e., harsh parenting). In a similar fashion, acknowledging that greater discrepancies in informant reports have been associated with worse child behavior outcomes (e.g., Gaylord et al. 2003; Ferdinand et al. 2004; Guion et al. 2009), we expect that discrepancy between parent-reported and observed *negative parenting* is larger when parents report higher levels of negative child behavior. We tested these hypotheses in a diverse at-risk sample of families by estimating restricted correlated trait–correlated (method – 1) [CT–C (M–1)] models (Geiser et al. 2008, 2012).

## Method

### Participants

Participants of the present study were 386 Dutch parent-child dyads participating in the ORCHIDS study (Weeland et al. 2017). Parents were aged between 23 and 51 years (*M*_*age*_ = 38.09, *SD*_*age*_ = 4.84, 91.00% mothers), and mostly born in The Netherlands (86% of mothers and 84% of fathers). Children were aged 4–8 years (*M*_*age*_ = 6.21, *SD*_*age*_ = 1.33, 55.30% boys) and showed mild to (sub)clinical externalizing behavior problems (see Table 1 for demographic characteristics of the sample). This age range was selected because it is a crucial age in the development of disruptive behaviors, and assessment of this behavior during this age period is essential for timely intervention (e.g., Prior et al. 2001).

**Table 1:** Demographic characteristics of sample (*N* = 386)

	*n*	*%*
Child’s gender (boys)	173	55
Parent’s gender (female)	356	91
No. of children in family (2 children)	223	58
No. of adults in family (2 adults)	338	87
Marital status parents (married)	279	72
Job occupation parents (employed)	285	74
Father’s education (HVE)	102	26
Mother’s education (HVE)	134	35

*HVE*, Higher Vocational Education (i.e., college/ applied sciences)

No. = Number

### Procedure

We acquired a diverse at-risk sample of families using the Eyberg Child Behavior Inventory (ECBI; Eyberg and Pincus 1999). Families were approached for participation via two Dutch regional health care organizations through a personalized information letter, including the ECBI to screen for children’s externalizing problem behavior. Children who scored at or above the 75th percentile of their respective cohort were invited to participate in the trial, resulting in an at-risk sample ranging from normal to clinical scores on externalizing behavior. Once included, parents were asked to fill out questionnaires on their own parenting behavior as well as their child’s behavior. Also, children and the participating parent were videotaped in a structured play situation during house visits. The videotaped situations were divided in four standardized five-minute episodes: free play; child-directed play; parent-directed play; and clean-up. The degree of parental control varied in each situation, for which parents received clear instructions. The first situation was meant for parents and children to get used to the observation setting: all parent and child dyads played with the same toys. The second situation was a child directed interaction (CDI), in which children were allowed to choose the toy and play according to their own rules. During the third situation, the parent-directed interaction (PDI), parents were instructed to lead the play situation based on their parental rules. The fourth situation was a clean-up (CU), where the parent was instructed to direct the child to put all the toys away without assistance. The instruments were selected based on their frequent usage in clinical practice and intervention research (e.g., Abrahamse et al. 2016; Leijten et al. 2017). All study procedures, information letters and methods were reviewed and approved by the central committee on research involving human subjects in The Netherlands (Medical Ethical Committee of the Utrecht Medical Centre (METC-UMC Utrecht protocol number 11–320/K). Together with the screening questionnaire, families received an information letter about the study and an active consent form. After screening, all families eligible for the study again received an information letter explaining the research procedure and goals. During the home visit a researcher or trained research assistant went through the main points of this letter and parents were given the opportunity to ask questions. Parents then signed informed consent for the collection and use of the observation and questionnaire data.

### Measures

#### Parent-Reports

##### Negative Child Behavior

For measuring parent-reported negative child behavior we used the Dutch version of the ECBI (Eyberg and Pincus 1999). The ECBI is a widely used 36-item parent rating scale to measure disruptive behavior problems in children (e.g., ‘Acts defiant when told to do something’). This inventory assesses the frequency (i.e., intensity) of the behavior using a 7 point Likert scale (1 = *never* to 7 = *always*). In order to reduce bias and optimize model fit, a latent score for negative child behavior was formed by assigning the items to three ECBI “subfactors” (i.e., parcels) (see Little et al. 2013). Parcels were created on the basis of those items that correlated highest with each other (Bandalos 2002). Reliability of the ECBI scores was satisfactory (Cronbach’s α = 0.85). Good validity was demonstrated (Abrahamse et al. 2015; Boggs et al. 1990). For example, the ECBI correlates with mother-reports of Conduct Problem and Hyperactivity/Impulsiveness scales of the Strenghts and Difficulties Questionnaire (*r* = .75) (Abrahamse et al. 2015). Also, significant differences on ECBI scores were found between a non-clinical sample of Dutch children (M_sumscore_ = 111.40) and a sample of Dutch children with conduct problems (M_sumscore_ = 162.32) (Abrahamse et al. 2015). 

##### Negative Parenting

Parenting behavior was measured by the Dutch version of the Parenting Practices Interview (PPI; Webster-Stratton 2001; Webster-Stratton et al. 2001), which is a 72-item questionnaire. The concept of *Negative Parenting* relates to parental use of harsh–inappropriate discipline, and encompasses seven items including spank–swat–whip, slap–hit, yell, and raise voice. PPI scores were rated on a 7-point Likert scale (1 = *never* to 7 = *always*). Latent scores were created on the basis of three PPI parcels for negative parenting respectively. Cronbach’s α for negative parenting was 0.79. Previous studies showed moderate to high stability of PPI scores over time (>.50 *r* <.77) (Baydar et al. 2003), as well as validity of the measure to assess changes in parenting behavior after intervention (Baydar et al. 2003; Weeland et al. 2017).

#### Observer-Reports

Observer-reported parenting and child behavior was acquired through the Dyadic Parent-Child Interaction Coding System (DPICS; Eyberg et al. 2005; Robinson and Eyberg 1981). This behavioral observation coding system has been extensively researched for recording behaviors of children and their parents during 20-min parent-child interactions (e.g., Shanley and Niec 2011). Convergent validity was demonstrated by the DPICS accounting for a significant proportion of variance in questionnaire scores on child problem behavior (ECBI Intensity scale: 45% of the variance), and questionnaires for parental locus of control (27% of the variance) and parenting stress (25% and 19% of the variance in child and parent domain respectively, Bessmer 1998). Discriminant analysis also showed the ability of the DPICS to classify families into clinic-referred and non-referred groups (i.e., the DPICS variables were found to classify correctly 94% of families into a clinic-referred or non-referred group, Bessmer 1998; Foote 2000). Moreover, clinical studies as well as intervention studies have extensively investigated child and parenting behaviors by the DPICS and found behavioral changes over time both in parenting behavior (Weeland et al. 2017) and child behavior (Posthumus et al. 2012).

The DPICS manual offers a standard coding procedure and coding sheet. The observations were coded by trained research assistants who were not involved in the study and who were blind to measurement wave. Monthly calibration meetings were held to prevent observer drift. To provide estimates of interrater reliability, a random 20% of the observations were independently coded by two coders. Coders were unaware of which observations were used to assess observer agreement.

The DPICS is often used as observation instrument to assess parenting and child behavior (e.g., Niec et al. 2015). However, there is little correspondence between studies in which DPICS categories for parent and child behavior are used. For instance, for child behavior some studies used a composite score including (Webster-Stratton et al. 2001) or excluding child non-compliance (Posthumus et al. 2012), whereas others used separate categories such as non-compliance or physical negative to index child externalizing behavior (Eyberg et al. 2001). Previous studies reported difficulties in forming reliable scales for this behavior (e.g., Weeland et al. 2017). Therefore, we carefully selected DPICS items in order to match the range of behaviors with the items of the parent questionnaires, and created latent scores in our CT–C (M–1) models, either using only DPICS items with acceptable factorloadings, or by creating parcels on the basis of inter-item correlations. To provide relevant descriptives and correlations of the latent variables in our CT–C (M–1) models, we formed composite scores that are based on the items and parcels that we included in the models (see Table 2).

**Table 2:** Descriptives of composite scores: minimum, maximum, means, standard deviations and correlations

	*M*	*SD*	*Min*	*Max*	1.	2.	3.	4.
1. Negative child behavior – observer report	9.30	9.87	0.00	55.00	–			
2. Negative child behavior – parent report	96.75	14.81	50.00	143.00	.06	–		
3. Negative parenting behavior – observer report	12.73	11.11	0.00	96.00	.36^**^	.10^*^	–	
4. Negative parenting behavior – parent report	33.95	7.58	15.00	54.00	.04	.28^**^	.12^*^	–

Composite scores are based on the items and parcels that were included in the CT-C(M - 1) models

Correlations are based on the square root transformed composite scores. Means and SDs are based on non-transformed composite scores

*p < 0.05, ** p < 0.01

##### Negative Child Behavior and Negative Parenting

For measuring observer-reported negative child behavior parcels were formed on the basis of inter-item correlations. Two items for non-compliance were taken together into one non-compliance parcel. The items cry, whine, yell, smart talk, and destructive were formed into a parcel oppositional behavior. The inter-observer reliability coefficient (intraclass correlation (ICC) using SPSS 22.0) for DPICS items of *Negative Child Behavior* was excellent: 0.92. For measuring observer-reported negative parenting the items critical statements, physical intrusion, and physical negative were taken as indicators (cf. Webster-Stratton et al. 2001). ICC for DPICS items of *Negative Parenting* (ICC = 0.80) was excellent.

### Data Analyses

The number of families included in the analyses was *N* = 386. In order to investigate parent-observer discrepancies on *parenting* and *child behavior* we performed two restricted  CT–C (M–1) models (Geiser et al. 2008, 2012) using version 20 of the AMOS program (Byrne 2013). We investigated parent-observer agreement on child and parenting behavior, where latent *regression* coefficients of > 0.10, > 0.30 and > 0.50 were interpreted as low, medium and high agreement respectively. Moreover, we investigated to what degree external variables of interest were related to parent-observer discrepancies, by testing two models: (1) *Negative Child Behavior* and (2) *Negative Parenting*. In the child behavior model, child gender and negative parenting were related to informant discrepancy. In the parenting model, child gender and negative child behavior were related to informant discrepancy.

In order to test our hypotheses on parent-observer informant discrepancies we used restricted CT–C (M–1) models (see Geiser et al. 2012). In contrast to the highly criticized traditional use of difference scores, methods such as polynomial regression and LD models have been proposed for examining informant discrepancies (Laird and De Los Reyes 2013; Laird and LaFleur 2016; Geiser et al. 2012). However, the latter methods are only suitable in the case of measurements on the same scale (e.g., parent-teacher reports from the same instrument). Geiser et al. (2012) provides us with a model especially useful for examining informant discrepancies when dealing with different measurement scales: the restricted CT–C (M–1) model. Restricted CT–C (M–1) models “provide useful insights into the ways in which methods might or might not differ from each other” (Geiser et al. 2012, p. 431). They allow to shed light on method effects by linking these effects to external variables, which is specifically desirable for answering our research question that concerns two different methods: an observation instrument versus a survey.

We followed Geiser et al. (2012) in their reasoning that the effect of the latent *regression* coefficient can be partialed out; a latent *residual* variable represents that part of a second method (i.e., *parent-report*) that cannot be predicted from a first reference method (i.e., *observer-report*). Geiser et al. (2012) suggest to choose the most valid or objective method as a reference method, based on theoretical considerations and ease of interpretation. Because of the systematic character of the observation instrument, and the trained research assistants that coded the observations, we chose the observer-report as a reference method. Importantly, the fit of the model does not change for different reference methods (Geiser et al. 2012). This latent *residual* variable was our key variable of interest, because it can be correlated with external variables to get more understanding of which factors are associated with this *residual* variable (correlations between the *residual* variable and external variables are *semipartial correlations*, see Geiser et al. 2012). The *semipartial correlations* tell us whether that part of the second method (i.e., *parent-report*) from which the influence of the reference method (i.e., *observer-report*) has been partialed out, is related to external variables.

In our case, the latent *residual* variables of interest were interpreted as the parent-observer discrepancies, and the standardized latent *regression* coefficients were interpreted as the level of agreement between parents and observers. The *semipartial correlations* between the parent-observer discrepancy variable and external variables –in our case reports of parenting or child behavior and gender– were our main interest, because they allow for correlation with the discrepancy variable. Specifically, the *semipartial correlations* shed light on informant discrepancies, since they can be easily related to the discrepancy variable in a CT–C (M–1) model.

**Fig. 1:**
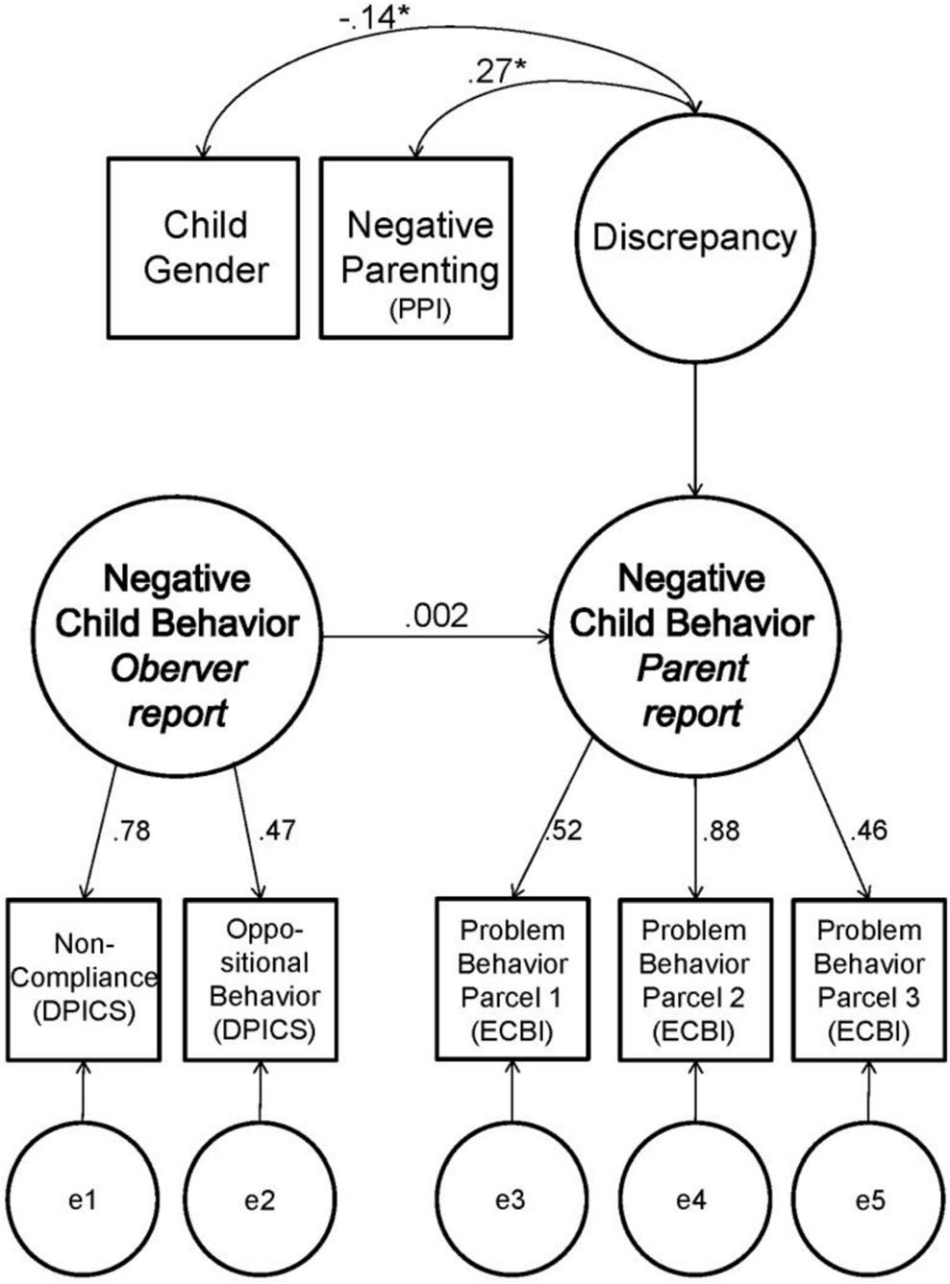
Negative child behavior restricted CT–C(M–1) model with standardized estimates

The models were tested by using the estimation method maximum likelihood (ML). Model fit was evaluated by means of the chi-square (χ^2^) statistic, Root Mean Square Error of Approximation (RMSEA), the Comparative Fit Index (CFI), and the Goodness of Fit Index (GFI). The chi-square test is a measure of exact fit. A significant chi-square value (*p* < 0.05) indicates that the model does not fit the data (Kline 2011). The RMSEA is a measure of approximate fit. RMSEA values of ≤ 0.08 (Hu and Bentler 1999; Kline 2011), and GFI and CFI between 0.95 and 0.97 are indicative for acceptable fit (Kline 2011). In order to fit the models correctly, assumptions were checked (one multivariate outlier was deleted) and “parcels” (Bandalos 2002) were created for both parent and observer-reports (see Figs. 1 and 3). DPCIS parcels in all models were square root transformed for normality. Parcels that showed factor loadings below 0.40 were eliminated from the models. Indicators showed that missing data was not problematic (less than 5% for all items). To replace missing data we used regression imputation, where cases with complete data are used to predict missing values for incomplete cases in a regression equation (Tabachnick and Fidell 2012, p. 68).

## Results

### Negative Child Behavior Model

In order to explain discrepancy between parent-reported and observed negative child behavior, we performed a restricted CT–C (M–1) model for negative child behavior. Figure 1 shows the negative child behavior model with its standardized estimates. The model shows acceptable fit, according to RMSEA, CFI and GFI measures of approximate fit, although the chi-square criteria for exact fit indicated no exact fit (see Table 3). However, it has been found that for models with larger sample sizes the chi-square is more often significant than with smaller sample sizes (see Kenny 2015).

The negative child behavior model represented almost no agreement between parents and observers in their reports of negative child behavior, indicated by the latent *regression* coefficient (*β* = 0.002, *p* = 0.980). The latent *residual* variable in this model was interpreted as the parent-observer discrepancy variable, and was related to negative parenting and child gender. As indicated by the *semipartial correlations*, parent-reported negative parenting appeared significantly related to the latent *residual* variable, that is, the parent-observer discrepancy variable (*r* = 0.27, *p* < 0.001). For the sake of clarity, from now on in this article the latent *residual* variable is directly referred to as *parent-observer discrepancy*.

**Fig. 2:**
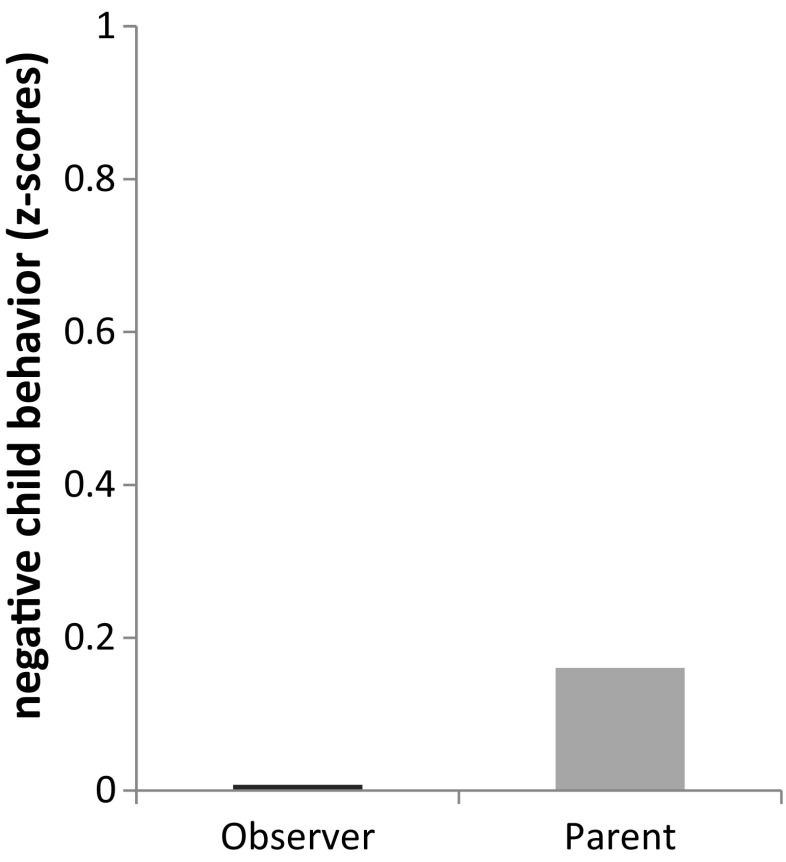
Illustration of observer and parent-reported *Z*-scores of negative child behavior for families where parents report high (i.e., above median) negative parenting

More specifically, more parent-reported negative parenting was related to higher parent-observer discrepancy on negative child behavior. For purpose of illustration only, Fig. 2 shows that in families where parents reported high negative parenting (based on a median split cut-off), parents reported higher levels of negative child behavior than were observed. This was in line with our hypothesis. Although small, we also found a significant relation for child gender with parent-observer discrepancy on negative child behavior (*r* = −0.14, *p* < 0.001). Parent-observer discrepancy was thus higher for boys than for girls. Additional analyses showed that in observer reports there was no significant difference between reports about boys and girls (*t* = −0.24, *p* = 0.81). In parent reports, boys were rated significantly higher than girls (*t* = 3.84, *p* < 0.001). This indicates that the parent report accounts for the variance between boys and girls in parent-observer discrepancy on negative child behavior.

### Negative Parenting Model

**Fig. 3:**
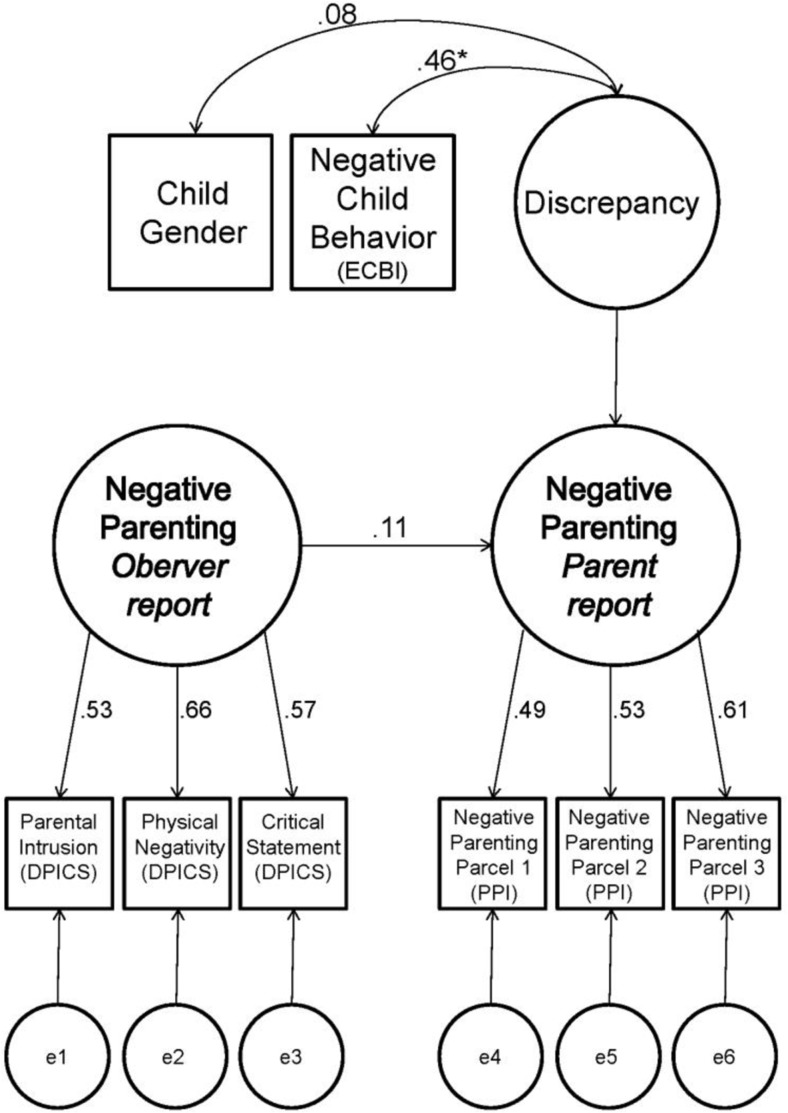
Negative parenting restricted CT–C(M–1) model with standardized estimates

In order to explain discrepancy between parent-reported and observed *parenting*, we performed a restricted CT–C (M–1) model for negative parenting. Figure 3 shows the negative parenting model with its standardized estimates. The model shows acceptable fit, according to RMSEA and GFI measures of approximate fit, although the 90% confidence interval of RMSEA slightly exceeds 0.08, and CFI values are below 0.95. The chi-square criteria for exact fit indicated no exact fit (see Table 3).

**Table 3:** Fit statistics for CT–C(M–1) models of negative child behavior and negative parenting

Model	*χ* ^*2*^ *(df)*	*p*	*RMSEA* [90% CI]	CFI		GFI
Negative child behavior	23.78 (14)	0.049	0.043 [0.003; 0.071]	0.960		0.983
Negative parenting	45.50 (19)	0.001	0.060 [0.038; 0.083]	0.906		0.972

*CI*, Confidence Interval

**Fig. 4:**
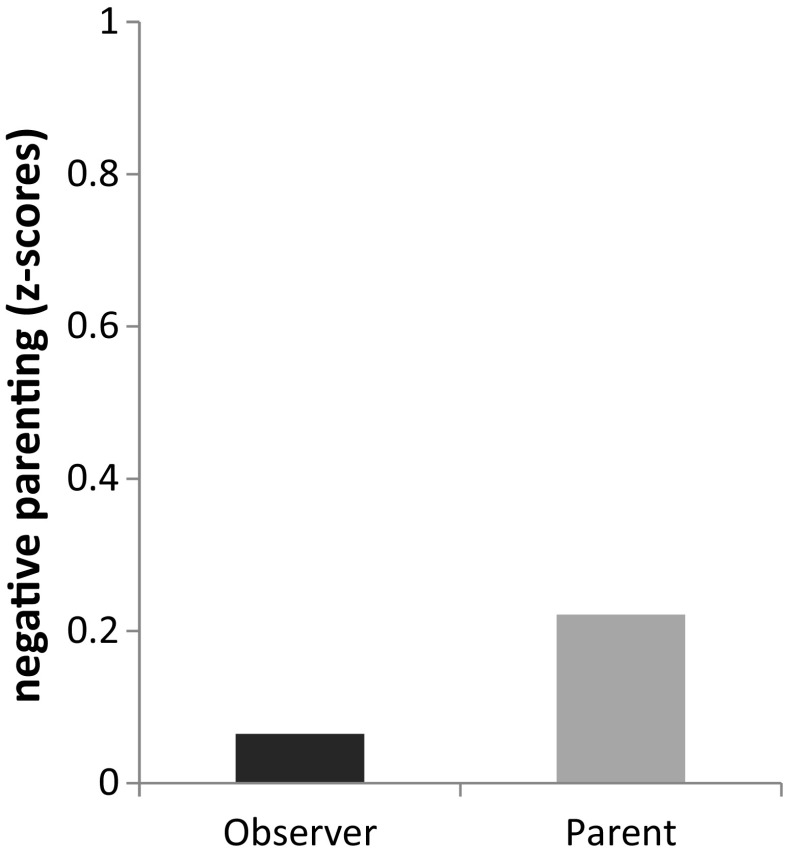
Illustration of observer and parent-reported *Z*-scores of negative parenting behavior for families where parents report high (i.e., above median) negative child behavior

The negative parenting model represented low agreement between parent-reported and observed negative parenting (*β* = 0.11, *p* = 0.170). In line with our hypothesis, parent-reported negative child behavior appeared significantly related to parent-observer discrepancy on negative parenting (*r* = 0.46, *p* < 0.001). In other words, parent-observer discrepancy in this model was related to parent-reported negative child behavior. More parent-reported negative child behavior indicated larger parent-observer discrepancy on negative parenting. For illustration purposes only, Fig. 4 shows that in families where parents reported relatively high levels of negative child behavior (based on a cut-off median split) parents reported higher levels of negative parenting than were observed. Child gender appeared to be not related to parent-observer discrepancy (*r* = 0.08, *p* = 0.197).

## Discussion

This study examined informant discrepancies between parent-reported and observed negative child behavior and negative parenting behavior (*N* = 386 parent-child dyads), as well as possible factors associated with these discrepancies. Informant discrepancies might be more than simply measurement error and might reveal important information on family functioning (see Hunsley and Mash 2007). Our results demonstrate high discrepancy (i.e. low agreement) between parents and observers on negative child behavior and negative parenting behavior, with parents scoring higher than observers. These findings on the magnitude of discrepancies are in line with previous research that demonstrated high to moderate discrepancies between different informants (Achenbach et al. 1987; Achenbach 2006; Duhig et al. 2000; Ferdinand et al. 2004; Guion et al. 2009; Hendriks et al. 2017; Sessa et al. 2001). Interestingly, these discrepancies probably represent more than simple disagreement, or different perspectives between informants. Discrepancies might direct us to negative family patterns, such as coercive parent-child interactions or parenting stress.

In this respect, a further endeavor of this study was to explore factors associated with parent-observer discrepancies, by correlating external variables to these discrepancies. Our results demonstrate that parent-reported *negative parenting* is positively related to discrepancy between parent-reported and observed negative child behavior (*r* = 0.27). When parents reported high levels of negative parenting behavior, higher discrepancy between parent and observer-reported negative child behavior was found. Although our data did not allow us to test actual underlying mechanisms, one possible explanation for this finding is that high levels of self-reported negative parenting behavior indicates high levels of parenting stress and low feelings of parental self-efficacy (Coleman and Karraker 1998; De Los Reyes and Kazdin 2005; Mash and Johnston 1983). For example, mothers of children with clinical behavior problems experience higher levels of parenting stress and less satisfaction in their parental role, than mothers of healthy children (Mouton and Tuma 1988). Parenting stress might predict parental irritability and criticism (Webster-Stratton 1990), and in turn a higher likelihood that parents report negatively on their own and their child’s behavior. Such negative parental perception biases have been related to adverse child outcomes such as delinquent behavior (De Los Reyes 2011; Ferdinand et al. 2004). In other words, high discrepancy between parent-reported and observed negative child behavior might indicate families in need of help. These discrepancies might be a proxy of underlying problems that have not been assessed with the instruments used and might for example point out those parents who experience high levels of stress and low levels of parental efficacy. This might be an indication that these families find themselves in a downward spiral and are particularly in need of intervention.

In addition, parent-reported *negative child behavior* is positively related to parent-observer discrepancy on negative parenting behavior (*r* = 0.46). When parents reported high levels of negative child behavior, high parent-observer discrepancy on negative parenting behavior was found. One possible explanation is that high parent-reported negative child behavior indicates parents’ irritability with their child’s behavior and in turn a bias in which they underestimate the reciprocal effect between their parenting behavior and the negative behavior of their child (e.g. Abidin 1992). Consequently, this allocation bias might result in a higher discrepancy between their own reports of negative parenting behavior and those of the observer. Although in our study the correlated variables were otherwise defined, our findings are in line with literature that successfully related discrepancies to (problematic) interaction patterns in families (see De Los Reyes 2011; De Los Reyes and Kazdin 2005; Guion et al. 2009; Harvey et al. 2013). Future research on informant discrepancies should take into account such measures of parent-child conflict, stress, behavioral attributions, and irritability to properly test these mechanisms.

Interestingly, child gender is related to parent-observer discrepancy on child behavior. Discrepancies on negative child behavior are higher for boys (*r* = −0.14). We specifically found that when parents rated behavior of boys, they generally reported higher levels of negative child behavior than were observed. In contrast, in the case of girls parents generally reported lower levels of negative child behavior than were observed. This might point to a bias in observers and/or parents: similar behaviors of boys and girls might be perceived differently between the sexes. Perhaps, observers evaluate behavior of girls differently from boys. Observer bias related to gender has been previously found in ratings of externalizing behaviors. Such “evaluative biases” might lie in the tendency to attribute socially desirable behavior to preferred participants (Waters and Deane 1985; Lyons and Serbin 1986). It could also be that parents are inclined to interpret boys’ behaviors fitting with a stereotypical notion of “externalizing” and acting out. Such an explanation is in accordance with other research, which suggested that parents show distortions in their perceptions of their own child related to their child’s gender. For example, child gender affects parents’ attributions for their children’s learning performance. Parents perceived learning performances differently for boys than for girls; they attributed boys’ performances to talent whereas girls’ performances were attributed to effort (Eccles et al. 1990; Räty et al. 2002). Practitioners should take into account such possible gender differences in parental or observer perceptions of child behavior. However, for parent-observer discrepancy on *parenting behavior*, child gender was not significantly related. In any case, future research should take into account these possible gender differences, because it has been found that different parenting behaviors are utilized for boys than for girls (McKee et al. 2007).

Although our results emphasize the importance of negative family dynamics as correlates of informant discrepancy, there may still be several other factors associated with informant discrepancies as well. First, given that relationships between parenting practices and child behavior are theorized to be bidirectional, the described mechanisms might also work the other way around (e.g., Abidin 1992; Burke et al. 2008; Patterson and Reid 1970). That is, negative bias caused by parenting stress or depression might precede conflictual parent-child interactions (instead of conflictual parent-child interactions causing bias; Haaga et al. 1991; Webster-Stratton 1990). Highly stressed parents might more easily behave in negative ways towards their children, driven by their negativity bias. In turn, this may lead to more conflictual parent-child interactions. Indeed, negative parental perception biases have been related to adverse child developmental outcomes (De Los Reyes 2011; Ferdinand et al. 2004, but see for a critical review Richters 1992).

Yet another explanation for informant discrepancy lies in the diversity of *contexts* in which different instruments for the assessment of parenting and child behavior were applied. On the one hand, parent-reports might inform us about child behavior across different parts of children’s days and routines, which might not be captured by observational procedures (Seifer 2005). In this respect, parent-reports might have a broader focus than observer-reports depending on visible child behavior at a specific time and in a specific context, possibly influencing discrepancies (see also Hartley et al. 2011). For example, the ECBI questionnaire asks parents to report how often behaviors such as temper tantrums and arguing occur *in general*, whereas with DPICS observations such behaviors are scored when they occur in the *specific moment* of observation. Although we matched the questionnaire items with the observation codings, it is likely that the observation only captures a subset of the total range of behaviors within parent-child interactions. Parents and children might act differently when they know they are being observed. Moreover, the playful nature of the tasks during the observation may have further limited negative behaviors during the observations. These factors may have confounded our findings and could have inflated the discrepancy between observer and parent reports. Particularly interesting would be to observe parent-child dyads in true ecological settings, for example by capturing real-life daily family routines on camera for a longer period of time (see Goldman et al. 2014), hereby providing a more complete view on parent-child interactions. Videotaping parent and child behaviors in different contexts, and over a longer time span, might create possibilities for observers to capture the broader view of daily family routines. For example, fixed cameras could be placed in families’ homes for a longer period of time. To our knowledge, commonly used observation systems (such as DPICS) have not been compared to such observations on real life settings. Having parents and observers report about the same time span and events might help clarify whether parent-observer discrepancies are context related or might be predicted by other factors.

Finally, some types of negative child behavior might be more easily captured by observational instruments than others. For example, out of the three dimensions of child behavior problems proposed by Stringaris and Goodman (2009), negative child behaviors categorized by emotional dysregulation and irritability might be more easily captured through observation than negative child behaviors that are categorized as callous (i.e., hurtful) or headstrong. Children might have more difficulties in suppressing behaviors caused by emotional dysregulation and irritability during observation. Such differentiation in negative child behavior might be important because it possibly indicates different etiology, comorbidity and prognosis, as well as intervention needs and responsiveness (Scott and O’Connor 2012; Stringaris and Goodman 2009). Future research could explore whether informant discrepancies can inform us on such different behavior patterns.

Several limitations of this study warrant mentioning. First, although we targeted at-risk families by screening them on externalizing child behavior (i.e., cut-off was 75th percentile of the ECBI), we ended up with a relatively well-off sample that consisted mostly of indigenous Dutch, well-educated parents and their children. This limits the generalizability of our findings to different samples and countries. Second, participants were mostly mothers (91%). Therefore, despite the fact that we used a sizeable sample we were unable to differentiate between fathers and mothers. Given the known gender differences in parenting strategies such as discipline and warmth (see McKee et al. 2007), this implies that our findings are not generalizable to father-observer discrepancies. It would be specifically interesting to replicate the gender effects found in our study among fathers. Third, although we complied with critique on the use of difference score models (Laird and De Los Reyes 2013) by choosing a suitable structural equation modelling (SEM) for examining informant discrepancies, our type of modelling has limitations as well (see Geiser et al. 2012). For example, the observer-perspective on child and parenting behavior was chosen as the reference for theoretical reasons –observer-instruments can be seen as a more systematic measure (Gardner 2000). However, one might argue this choice is arbitrary since there is no consensus on a “gold standard” (Richters 1992). Fourth, although the use of item parcels in SEM has become commonly accepted for unidimensional constructs, this procedure has also been critiqued by some. For example, it could mask a multidimensional factor structure of the construct, causing unreliable fit indexes for the specified model (i.e., good fit for a misspecified solution, Bandalos 2002). Fifth, the ecological validity of observational instruments like the DPICS have been questioned (Wakschlag et al. 2010). Although the DPICS was shown to be a valid measure of parenting and child behavior (Bessmer 1998; Foote 2000), it remains unclear whether short, pre-structured interaction episodes can result in ecologically valid measures of negative child behavior and parenting behavior (see also Weeland et al. 2017). Finally, since we compared an observational instrument to questionnaires, with items not exactly interchangeable, this may have led to differences related to the type of instrument. Discrepancy levels may have been influenced by the nature of the observation codings relative to what was asked in the questionnaires.

Despite these limitations our study contributes to the literature in several ways. First, to our knowledge this is the first study investigating discrepancies linking observers’ data to parent-reported survey data on child externalizing behavior. Until now, the growing body of research on factors contributing to multi-informant discrepancies mostly focused on discrepancies between mothers, fathers, teachers, and children (e.g., Harvey et al. 2013). Most importantly, our study features a novel empirical effort to identify associations with informant discrepancies, rather than dismissing these as measurement error or “noise”. We did this by using a sophisticated, SEM-based analytical strategy in a large, relevant sample of children at-risk for the development of externalizing problem behavior. Previous studies on associates of informant discrepancies mostly focused on predictors such as socioeconomic factors (Duhig et al. 2000; Stone et al. 2013). Our results indeed indicate that reports of parenting and child behavior are associates of parent-observer discrepancies. This suggests that discrepancies are informative and meaningful in itself because they relate to family functioning. In this way, information on discrepancies can reveal more understanding of families, and taking into account this meaningful information can be helpful in clinical practice.
